# The World Congress of Insulin Resistance, Diabetes and Cardiovascular Disease (WCIRDC)

**DOI:** 10.1111/1753-0407.13349

**Published:** 2023-01-06

**Authors:** Zachary T. Bloomgarden

**Affiliations:** ^1^ Department of Medicine Mount Sinai School of Medicine New York New York USA

The 20th Annual World Congress of Insulin Resistance, Diabetes and Cardiovascular Disease (WCIRDC) offered an overview of a variety of aspects of diabetes pathogenesis and complications. An initial series of presentations was given as a tribute in memory of Gerald Reaven, who was responsible with Yehuda Handlesman for the initial meeting of the WCIRDC in 2002. Lawrence Mandario presented concepts of the role of the thermodynamic state of skeletal muscle in regulating insulin sensitivity, suggesting a relationship between muscle mitochondrial content and insulin sensitivity^1^ and an effect of insulin in increasing muscle energy expenditure not seen among persons with type 2 diabetes. Philipp Scherer revisited the notion that excess ligand stimulates resistance to the action of that ligand in a fashion that might also be true for insulin, with hyperinsulinemia triggering insulin resistance even in the absence of hyperglycemia^2^ and with the adipocyte central in the control of systemic insulin sensitivity.^3^ An implication is that high doses of insulin may indeed contribute to insulin resistance and hepatic steatosis, perhaps explaining aspects of the therapeutic benefits of the glucagon‐like peptide (GLP)‐1 agonists and sodium‐glucose cotransporter‐2 (SGLT2) inhibitors. Steven Kahn discussed interrelationships between insulin action and insulin secretion, pointing out that modeling approaches based on peripheral insulin levels need to take into account hepatic insulin clearance,^4^ which leads to the conclusion that diabetes does not develop until 90% of insulin secretion is lost. In the ADOPT (A Diabetes Outcome Progression Trial) study, progressors to monotherapy failure had lower baseline insulin secretion than patients who were successfully treated with a single agent, although having levels of insulin resistance similar to that in those who responded to monotherapy.^5^ Furthermore, genes associated with beta cell dysfunction track with type 2 diabetes development,^4^ leading Kahn to conclude that “the beta cell seems to be the critical component.” Insulin resistance, however, appears to track particularly well with cardiovascular disease (CVD) outcomes, with Ralph DeFronzo reviewing the large number of studies of this relationship, perhaps with a defect in insulin signaling at the level of insulin receptor substrate 1 leading to decreased activation of the GLUT4 glucose transporter and of nitric oxide synthase, while activation increases of the mitogen‐activated protein kinase pathway leading to inflammation, atherosclerosis, and vascular smooth muscle proliferation.^6^ A striking example of this relationship was shown in one of Dr Reaven's last publications, a study of 1720 men initially evaluated at age 50 in 1970, where those with the highest quartile of the triglyceride/high‐density lipoprotein (TG/HDL) cholesterol ratio both had insulin resistance and, at 40‐year follow‐up, a 47% increase in likelihood of myocardial infarction and/or stroke.^7^

Tracey McLaughlin discussed the association of obesity with adverse COVID‐19 outcome, noting the presence of angiotensin‐converting enzyme‐2 (ACE‐2) in adipose tissue appearing to be expressed by specific macrophages, which then act as receptors for SARS‐CoV‐2 viral entry.^8^ COVID infects and replicates in subcutaneous, epicardial, and paracardial adipose tissue beds, suggesting this to be a major site of ongoing viral infection. Linking to insulin resistance, McLaughlin discussed evidence that the TG/HDL ratio prior to COVID is a predictor of severe acute COVID and an independent predictor of “long COVID.” Pam Taub expanded on the long COVID syndrome, which may affect as many as one third of persons who have had COVID, with a wide variety of symptoms, including fatigue, decline in quality of life, muscular weakness, joint pains, dyspnea, cough, anxiety/depression, sleep/cognitive disturbances (so‐called “brain fog”), headaches, palpitations, and chest pain.^9^ She described the recently recognized association of long COVID with the Postural Orthostatic Tachycardia Syndrome (POTS) of excess sympathetic nervous system activity post viral illness, with orthostatic hypotension associated with tachycardia, rather than the typical bradycardia of vasovagal orthostatic hypotension.^10^ Taub speculated that orthostatic decrease in cerebral blood flow could explain some of the cognitive abnormalities of long COVID and commented on the use of ivabradine, which acts at the sinoatrial node and can preferentially reduce rapid heart rate, with a study of its potential role in long COVID currently ongoing. It is intriguing to recognize that orthostatic hypotension and cardiac autonomic neuropathy are associated with measures of insulin resistance,^11^ suggesting a potential mechanism of long COVID to involve decreased insulin sensitivity.

John Kirwan discussed an important component of insulin resistance beyond the excess in adiposity, that of sarcopenic obesity, with approximately 15% of persons with obesity having sufficient reduction in muscle mass and/or function to satisfy criteria for disability. Furthermore, sarcopenia is associated with increased mortality in patients with CVD undergoing surgery.^12^ Paradoxically, weight loss treatment is typically associated with reduction in muscle mass, potentially lessening the health benefit of intervention. Kirwan reviewed studies of a small molecule mitochondrial uncoupler reducing fat mass and improving insulin sensitivity while increasing muscle mass and function in animal models, offering a potential avenue of approach.^13^

Christos Mantzoros received the Leader in Insulin Resistance Award of the WCIRDC, quoting Homer's introduction to the Odyssey describing “twists and turns [and] far journeys,” with research in the complexities of adipose tissue in producing leptin, with deficiencies seen in hypothalamic amenorrhea and in lipodystrophy,^14^ in producing follistatin,^15^ having autocrine effects on release of follicle‐stimulating hormone; on bone, muscle, liver, and in mediating reduction in insulin sensitivity; and in producing the insulin sensitizer adiponectin,^16^ which may underlie the benefits of thiazolidinediones.

Richard Pratley discussed pancreas exocrine‐endocrine interrelationships, pointing out that although the islets comprise <2% of pancreatic mass, persons with type 1 diabetes have decreased overall pancreatic size. Although there is lesser decrease in size of the pancreas in type 2 diabetes, the fat content of the pancreas is increased, and pancreatic fat content is associated with the degree of insulin resistance.^17^ Diabetes remission following weight loss intervention is associated reduction in intrapancreatic fat and with normalization of the morphology of the pancreas,^18^ additional evidence of the importance of this association. Pancreatitis is associated with diabetes risk and diabetes with pancreatitis risk, and post‐pancreatitis diabetes is associated with greater risk of pancreatic cancer than type 2 diabetes,^19^ suggesting further complications of what is being termed “pancreatogenic diabetes,” or “type 3c diabetes.”^20^

Sonia Caprio began a set of talks on the progression from childhood risks to diseases in adults, describing her studies of the four classes of obese youth with progressively worsening glucose tolerance, in association with hyperinsulinemia, decreased insulin sensitivity, decreased insulin clearance, and increased liver fat.^21^ Petter Bjornstad reviewed an analysis of 517 persons with youth‐onset type 2 diabetes followed from 2011, at mean age 14, for an average 7.5 years, with HbA1c increasing from 6.0% to 9.3%; both hyperfiltration and insulin resistance tracked with development of microalbuminuria in 55% of the group, with an additional 17% developing hyperfiltration (estimated glomerular filtration rate ≥ 135).^22^ A study of 161 Pima Indians with type 2 diabetes who underwent renal biopsy showed that, compared with adult‐onset, youth‐onset diabetes was associated with greater levels of albuminuria and with greater glomerular basement membrane width and mesangial fractional volume independent of historic A1c control, age, and duration, with the youth‐onset group having greater risk of progression to kidney failure.^23^ Based on these findings, Bjornstad noted that SGLT2 inhibitors are being used at his center in treatment of youth with evidence of diabetic nephropathy. Alan Sinaiko described the International Childhood CV Cohort (i3C) studies in which determinants of fatal CV events at mean age 47 were assessed in 38 589 participants; for each SD of increase in combined risk score based on body mass index, systolic blood pressure, total cholesterol level, TG level, and smoking at age 12, there was a nearly threefold increase in mortality.^24^ There has, however, been little improvement in control of diabetes, lipids, or blood pressure.^25^ “It is time,” Sinaiko said, “to develop effective public health strategies to prevent early CV disease, CV events, and type 2 diabetes.”

Sinaiko's theme was taken up by Yehuda Handelsman in a talk on early intervention for patients with diabetes and cardiorenal and metabolic diseases. Handelsman discussed the “void in understanding the timing, sequence, and intensity of the management of patients with obesity, diabetes and comorbidities.” There is evidence that cardiometabolic health in the United States worsened from 1999 to 2018, primarily driven by increasing levels of obesity,^26^ and Handelsman suggested that there may be a point after which diabetes, hypertension, and renal insufficiency become irreversible, supporting the need to “manage disease early.” He reviewed a study comparing patients with HbA1c below 7% who received intensive treatment before 1 year after diagnosis with those having HbA1c ≥ 7% not receiving such treatment, the latter group having 67%, 64%, and 51% increase in risk of myocardial infarction, heart failure, and stroke, respectively.^27^ Another study of newly diagnosed persons with type 2 diabetes showed that those attaining HbA1c < 7.5% within the first year from levels >9% had nearly 40% fewer CVD events than those remaining in poor control.^28^ Thus, for obesity, metabolic syndrome, prediabetes and diabetes, hypertension, dyslipidemia, nonalcoholic steatohepatitis, obstructive sleep apnea, renal disease, neuropathy, retinopathy, and not least heart failure and atherosclerotic CVD. Handlesman expanded on Sinaiko's message, pointing out that we must on an individual patient basis as well as on a public health level intervene early to reduce adverse health outcomes.
